# Assessment of the ability to predict complications of the Risk Factor Scale for Pre-eclampsia Complications and the fullPIERS scale in pregnant women in a Hospital in Lima, Peru

**DOI:** 10.17843/rpmesp.2025.421.14041

**Published:** 2025-02-19

**Authors:** Patricia N. Aquino-Vásquez, Luis GM. Chuquipoma-Zanabria, Maria Lazo-Porras, Mónica Flores-Noriega

**Affiliations:** 1 Escuela de Medicina, Universidad Peruana Cayetano Heredia, Lima, Peru. Escuela de Medicina Universidad Peruana Cayetano Heredia Lima Peru; 2 CRONICAS, Centro de Excelencia en Enfermedades Crónicas, Universidad Peruana Cayetano Heredia, Lima, Peru. CRONICAS Centro de Excelencia en Enfermedades Crónicas Universidad Peruana Cayetano Heredia Lima Peru; 3 Clínica Angloamericana. Lima, Peru. Clínica Angloamericana Lima Peru; 4 Cayetano Heredia Hospital, Lima, Peru. Hospital Cayetano Heredia Lima Peru

**Keywords:** Preeclampsia, Pregnancy complications, Pregnancy

## Abstract

**Objectives.:**

To evaluate the ability of the Risk Factor Scale for Preeclampsia Complications (RFSPC) and the fullPIERS (Pre-eclampsia Integrated Estimate of RiSk) scale to predict complications of preeclampsia in pregnant women diagnosed with preeclampsia who were admitted to the obstetrics and gynecology department of a referral hospital, from October 2021 to December 2022.

**Materials and methods.:**

This was a retrospective cohort design study. Data was collected from the medical records of patients diagnosed with preeclampsia, and both scales (RFSPC and fullPIERS) were applied. With these results, the sensitivity, specificity and the area under the ROC curve (AUC) were obtained by taking different cut-off points. The best score was selected as the one with the highest AUC. The differences between the scales were explored by comparing their AUCs.

**Results.:**

We included 367 pregnant women. The RFSPC had a sensitivity of 71%, a specificity of 73% and an AUC of 0.722 with a cutoff point of 3 points. Whereas the fullPIERS scale showed 76%, 84% and 0.804 respectively with a cutoff point of 0.75%.

**Conclusions.:**

Both scales can be useful for identifying pregnant women at risk of complications with cutoff points different from those defined internationally.

## INTRODUCTION

Preeclampsia is a progressive multisystemic disease and is one of the hypertensive disorders that may occur during pregnancy. Physio-pathologically, preeclampsia has two stages. The first stage is characterized by abnormalities in uterine vascularization in the first trimester, leading to abnormal placentation and placental ischemia. It is in this stage that angiogenic factors, which are key proteins in the process of new blood vessel formation, fundamental for tissue growth, development, and repair, are implicated. One of the most relevant factors is the vascular endothelial growth factor, which promotes the proliferation and migration of endothelial cells, and platelet-derived growth factor, which plays a crucial role in stabilizing newly formed vessels. The release of these factors causes systemic endothelial dysfunction, marking the onset of the second stage, which manifests as acute vascular damage and/or hypoperfusion ^1^.

According to the American College of Obstetricians and Gynecologists (ACOG), preeclampsia is diagnosed based on the recent onset of high blood pressure (HBP) after 20 weeks of pregnancy, along with proteinuria. However, there are other clinical manifestations such as thrombocytopenia, renal failure, pulmonary edema, liver disease, visual disturbances, or right upper quadrant pain, which, when present in conjunction with HBP, are sufficient to establish the diagnosis of preeclampsia with criteria for severity ^2^^,^^3^. Hypertensive disorders of pregnancy affect 10% of pregnancies worldwide. In developed countries, complications from anesthesia and cesarean sections are the leading cause of maternal death, while in Latin America, preeclampsia is the leading cause, accounting for 25.7% of deaths in 2011. The incidence of severe preeclampsia varies from 2-5% in developed countries to up to 18% in developing countries ^4^^,^^5^.

Due to the serious consequences of preeclampsia on maternal and fetal health, it is crucial to have a scale with reliable parameters to predict its complications early, which include maternal death, eclampsia, acute cerebral events, pulmonary thromboembolism, hepatic hematoma, acute kidney injury, coagulopathies, and severe thrombocytopenia ^6^^-^^8^. Studies, such as the PETRA trial ^9^, have been conducted to predict adverse outcomes in pregnant women with preeclampsia. This trial compared management strategies in cases of early severe preeclampsia and their impact on the mother and fetus, which allowed the development of scales to predict severe complications of this disease. The fullPIERS scale ^10^^,^^11^, developed between 2003 and 2010, is a risk prediction model for preeclampsia based on data from more than 2,000 women. It has a sensitivity rate of 76% and a specificity rate of 87% for predicting adverse maternal outcomes within 48 hours of diagnosis. It evaluates six criteria and can predict complications up to seven days in advance. Although the objective is for it to be implemented worldwide, its development in high-income countries could affect its applicability in Latin America.

Another scale was developed in Mexico by Elizalde *et al*. in 2014, called the Risk Factors for Preeclampsia Complications Scale (EFRCP). It was developed based on a case-control study of patients with preeclampsia who did or did not experience complications ^12^. Risk factors capable of forming part of an instrument that predicts complications of preeclampsia were obtained. The scale has 12 parameters and has a sensitivity rate of 93% and specificity rate of 80% ^13^. It has been applied exclusively in the country where it was designed, Mexico, but it hasn’t been evaluated in other countries so far.

The EFRCP and fullPIERS scales can be useful in decision-making for the early treatment of preeclampsia, with the aim of reducing negative consequences for both the mother and the fetus. Given that preeclampsia is one of the leading causes of maternal and fetal mortality in developing countries, the use of these scales is justified to identify pregnant women at higher risk of complications and provide timely medical care. This research aimed to evaluate the predictive ability of preeclampsia complications using the EFRCP and fullPIERS scales in patients diagnosed with preeclampsia in a hospital in Lima during the period from October 2021 to December 2022.

**KEY MESSAGES**

Motivation for conducting the study. Preeclampsia is a significant cause of maternal and fetal complications. There is no standardized use of any tool to improve early identification and optimize treatment of pregnant women with preeclampsia in Peru.

Main findings. Both scales effectively predicted preeclampsia complications at their optimal cut-off points.

Implications. These predictive scales provide evidence for future research essential to reducing complications and their maternal-fetal repercussions associated with preeclampsia.

## MATERIALS AND METHODS

### Study design and context

This was a retrospective cohort study conducted at the Cayetano Heredia Hospital, a level III-1 hospital located in northern Lima, Peru. The area covered by this hospital has a population of 3,143,582 inhabitants, who generally have limited economic resources and are affiliated to the Comprehensive Health System (SIS) ^14^^,^^15^. In addition, according to reports from the Obstetrics Department, in 2019 there were almost 5,000 births per year.

### Population

We included pregnant women diagnosed with preeclampsia who were admitted to the Obstetrics Department of Cayetano Heredia Hospital from October 2021 to December 2022. The patients included in the study are pregnant women from the 20th week of gestation onwards with the criteria for the diagnosis of preeclampsia ^16^: High blood pressure determined by systolic pressure ≥140 and/or diastolic pressure ≥90 on two occasions at least 4 hours apart after week 20 of pregnancy in a woman with previously normal blood pressure, systolic pressure ≥160 and/or diastolic pressure ≥110 mm Hg (considered severe hypertension) proteinuria: ≥300 mg in 24-hour urine, protein/creatinine ratio ≥0.3 mg/dl, or test strip reading 2+. In the absence of proteinuria, the diagnosis was considered when the patient developed new-onset gestational hypertension and met any of the following criteria: Thrombocytopenia (platelet count ≤100,000 x 10*9/L), renal failure (serum creatinine >1.1 mg/dL or doubling of serum creatinine in the absence of renal disease), impaired liver function (transaminases twice the normal concentration), persistent severe pain in the upper right quadrant or epigastric pain without an alternative diagnosis, pulmonary edema, headache that does not respond to medication and cannot be explained by an alternative diagnosis, visual symptoms (blurred vision, lights, scotomas, flashes).

Patients excluded from the study were those diagnosed with preeclampsia who also had the following diagnoses: Maternal death, eclampsia, cerebrovascular event, pulmonary edema, pulmonary thromboembolism, HELLP syndrome, hepatic hematoma, acute kidney injury (AKI) II or III, dialysis, severe thrombocytopenia, coagulopathy, obstetric hemorrhage, refractory hypertension.

### Sample

A virtual calculator was used to determine the sample size ^17^^,^^18^. The sensitivity and specificity values of the fullPIERS scale were used, which are 76% and 87%, respectively ^8^. The prevalence of complications was set at 20%, which corresponds to the prevalence of eclampsia reported by a previous study ^19^. With an accuracy of 10% and a confidence level of 95%, the sample size was calculated at 351 patients.

### Operational definition of variables

In this study, the outcome variable is maternal complications of preeclampsia, while the predictor variables are the results of the fullPIERS scale and the results of the EFRCP.

The definitions and measurements of each one are explained below: Complications of preeclampsia are complications arising from hypertensive disease of pregnancy or preeclampsia. The information was obtained from medical records. In the study, the value “yes” corresponds to the presence of at least one of the following complications: coagulopathy, eclampsia, pulmonary edema, obstetric hemorrhage requiring surgical intervention or blood products, difficult-to-control hypertension, acute kidney injury, HELLP syndrome, or maternal death.

The fullPIERS scale is a tool designed to predict the onset of preeclampsia complications within 48 hours of hospitalization in a patient diagnosed with preeclampsia. However, the scale showed favorable results in predicting complications even within the first 7 days after hospitalization (AUC >0.7). The variables it considers are: gestational age, chest pain or dyspnea, oxygen saturation, platelet count, serum creatinine, and serum aspartate aminotransferase (AST) measured at the time of the patient’s hospitalization. These values are entered in the official scale calculator ^9^, and the result is expressed as a percentage indicating the probability of developing one or more complications of preeclampsia. The cutoff point determined to consider a patient at high risk for developing one or more complications of preeclampsia is ≥30%, with a sensitivity of 75.5%, specificity of 86.9%, and an area under the ROC curve (AUC) of 0.88. The relevant concepts for each of the variables in the FullPIERS Scale are detailed in Supplementary Material Appendix 1.

The Preeclampsia Complication Risk Factor Scale (EFRCP) is a tool that allows the prediction of preeclampsia complications. It is a scale that was developed and validated in Mexico ^12^. Twelve parameters were determined during the development of the scale. These are: maternal age, gestational age at diagnosis of preeclampsia, severe headache, dyspnea, platelets, mean platelet volume, INR, serum creatinine, serum uric acid, glutamic oxaloacetic transaminase, and serum lactate dehydrogenase. Each variable was assigned a score; finally, the total sum of the scores for the positive variables determined the final score on the scale. Thus, an action was assigned according to the score obtained on the scale: 0 points ruled out complications; 1-2 points required follow-up by a physician and indicated a low probability of complications; 3 points meant that the patient should be evaluated and monitored by intensive care personnel without the need for admission to the intensive care unit. Finally, 4 points or more meant a high risk of complications and admission to the intensive care unit was recommended. This cut-off point had the following statistical values: sensitivity: 93%, specificity: 80%, and AUC: 0.98. The relevant concepts for each of the variables on the EFRCP scale are detailed in Annex 2.

Sociodemographic variables were included to describe the characteristics of the population. These are maternal age (<18, 18-34, ≥35), country of origin (Peru, Venezuela, Colombia), gestational age (≤27, 28-36, ≥37), number of pregnancies (1, ≥2), parity (0, ≥1), and fetal sex (male, female).

### Procedures and techniques

The medical records of pregnant women admitted to the emergency department were reviewed weekly, and those who met the inclusion and exclusion criteria were identified for convenience. A data collection form was created and filled in with the patients’ clinical and demographic data, diagnostic criteria for preeclampsia, data to verify complications with their respective diagnostic criteria, data on the predictive variables of the fullPIERS calculator, data on the variables of the EFRCP, as well as data on the date of application of the form, hospitalization, discharge, death, delivery, and diagnosis of complications. The form was filled out and stored on the Google Forms virtual platform, which was accessible only to researchers.

### Statistical analysis

Initially, the important sociodemographic and obstetric variables were described, reporting categorical variables in frequencies and percentages. The database was analyzed using RStudio, an individual analysis of each scale was performed, and the best cutoff point for classifying a patient as high risk for complications was determined. To do this, each scale was examined with different cutoff points. To select these cut-off points, the first step was to find the cut-off point with the best AUC. Once this was found, the other points were selected based on their proximity to the cut-off point in the original study and in our study. In the case of the fullPIERS scale, the following values were taken as cut-off points: 30%, 10%, 5%, 1%, 0.75%, and 0.5%. In the case of the EFRCP scale, the values ≥1, ≥2, ≥3, ≥4, and ≥5 were taken as cut-off points.

The diagnosis of preeclampsia complications was used as the gold standard to evaluate the diagnostic performance of the scales. Sensitivity, specificity, positive predictive value (PPV), negative predictive value (NPV), positive likelihood ratio (PLR+), negative likelihood ratio (PLR-), and AUC were calculated. Diagnostic performance was evaluated considering the presence or absence of complications. To find the best cutoff point, the point or value with the highest sensitivity and specificity given by the AUC was selected.

The diagnostic performance of the fullPIERS calculator and the EFRCP scale was compared using the AUC and a proportion test (DeLong test). Given the lack of the “uric acid” parameter in the EFRCP scale, a parallel analysis was proposed including this parameter as positive for all, and the differences with the study without uric acid were explored; subsequently, the analysis with the best diagnostic performance was chosen to be compared with the fullPIERS scale.

### Ethical considerations

This study was approved by the ethics committees of Cayetano Heredia University and Cayetano Heredia Hospital to ensure that the project complies with Good Research Practice standards.

## RESULTS

**Figure 1:**
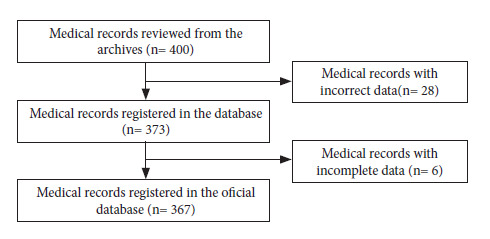
Participant selection flowchart.

Four hundred medical records were reviewed, of which 28 were discarded due to erroneous data. We entered 373 records into the database, 6 of which had incomplete data, so finally 367 records were entered into the official database, which was exported to Excel format (Figure 1).

### Characteristics of the study population

We found that 1.9% of pregnant women in the sample were adolescents and 25.6% were of advanced maternal age. Besides, 22.6% were of foreign origin. (Table 1) and 6% (21 participants) of the patients had some complication of preeclampsia. The two most common complications were obstetric hemorrhage requiring surgical intervention or blood products and difficult-to-control hypertension, with five cases each.

**Table 1:** Descriptive analysis of the population included.

Characteristics		n (%)
Maternal age		
	<18	7 (1.9)
	18 - 34	266 (72.5)
	≥ 35	94 (25.6)
Country of origin		
	Peru	284 (77.4)
	Venezuela	82 (22.3)
	Colombia	1 (0.3)
Gestational age		
	≥ 37	261 (71.1)
	36-28	103 (28.1)
	≤27	3 (0.8)
Number of pregnancies		
	1	107 (29.2)
	≥2	260 (70.8)
Parity		
	0	141 (38.4)
	≥1	226 (61.6)
Sex of the fetus		
	Male	194 (52.9)
	Female	173 (47.1)
Complications		
	Yes	21 (5.7)
	No	346 (94.3)
Type of complications		
	Coagulopathy	2 (0.5)
	Eclampsia	2 (0.5)
	Pulmonary edema	1 (0.3)
	Obstetric hemorrhage requiring surgical intervention or blood products	5 (1.3)
	Difficult-to-control hypertension	5 (1.3)
	Acute kidney injury	3 (0.8)
	HELLP syndrome	2 (0.5)
	Maternal death	1 (0.3)

### fullPIERS scale analysis

**Figure 2:**
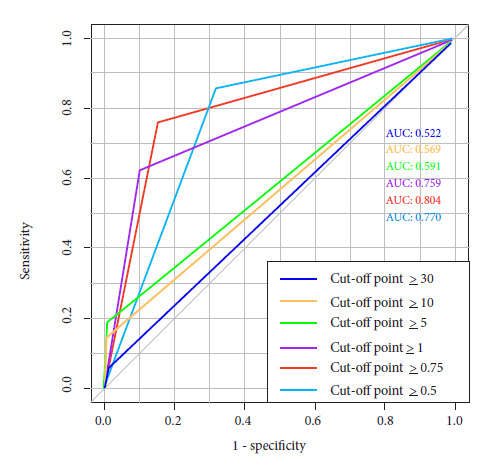
FullPIERS scale: Comparison of cut-off points.

We determined that the best cut-off point was 0.75%, yielding the following results: Sensitivity: 76%, specificity: 84%, LHR + 4.9 LHR - 0.3, PPV: 23%, NPV: 98%, AUC: 0.804 (Figure 2). The results of the different cut-off points are shown in Table 2.

**Table 2:** Cut-off points and parameters evaluated on the fullPIERS scale.

Cut-off point	Sensitivity (95% CI)	Specificity (95% CI)	LHR (95% CI)	PPV	NPV	AUC
≥ 30	4.8% (0 - 23)	99.7% (98 - 99)	+: 16.5 (1.1 - 254.2) -: 0.9 (0.86 - 1.05)	50.0%	94.5%	0.522
≥ 10	14.3% (0.3 - 36)	99.4% (97 - 99)	+: 24.7 (4.4 - 139.9) -: 0.9 (0.7 - 1.0)	60.0%	95.0%	0.568
≥ 5	19.1% (0.5 - 41)	99.1% (97 - 99)	+: 22.0 (5.2 - 91.8) -: 0.8 (0.6 - 1.0)	57.1%	95.3%	0.591
≥ 1	61.9% (38 - 81)	89.9% (86 - 92)	+: 6.1 (3.8 - 9.7) -: 0.4 (0.2 - 0.7)	27.1%	97.5%	0.759
≥ 0,75	76.2% (52 - 91)	84.6% (80 - 88)	+: 5.0 (3.5 - 7.0) -: 0.3 (0.1 - 0.6)	23.2%	98.3%	0.804
≥ 0,5	85.7% (63 - 96)	68.2% (63 - 73)	+: 2.7 (2.1 - 3.4) -: 0.2 (0.1 - 0.6)	14.1%	98.7%	0.769

AUC: Area under the curve, 95% CI: 95% confidence interval, LHR: Likelihood ratio, PPV: Positive predictive value, NPV: Negative predictive value

### Analysis of Risk Factor Scale for Preeclampsia Complications

**Figure 3:**
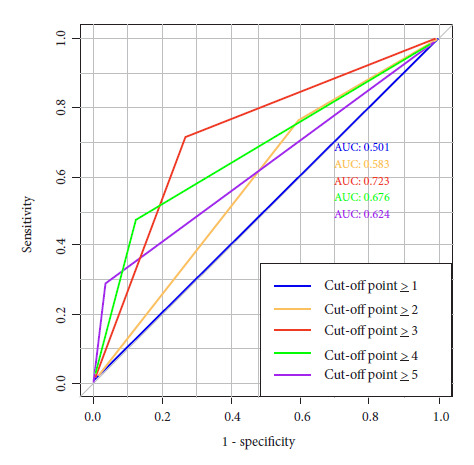
EFRCP scale without uric acid: Comparison of cut-off points.

The analysis determined that a cutoff point of 3 was the best value for considering a patient to be positive according to the scale, yielding the following results: sensitivity: 71%, specificity: 73%, LHR+ 2.6, LHR- 0.3, AUC: 0.722 (Table 3 and Figure 3).

**Table 3:** Cut-off points and parameters evaluated in the Risk Factor Scale for preeclampsia complications without uric acid.

Cut-off point	Sensitivity (95% CI)	Specificity (95% CI)	LHR (95% CI)	PPV	NPV	AUC
≥1	100% (84 - 100)	0% (0 - 1)	+: 1.0 (0.9 - 1.0) -: 0.0	5.7%	100%	0.501
≥ 2	76.2% (52 - 91)	40.4% (35 - 45)	+: 1.3 (0.9 - 1.6) -: 0.6 (0.3 - 0.3)	7.2%	96.5%	0.583
≥3	71.4% (47 - 88)	73.1% (68 - 78)	+: 2.7 (1.9 - 3.6) -: 0.4 (0.1 - 0.7)	13.8%	96.7%	0.723
≥ 4	47.6% (25 - 70)	87.6% (83 - 90)	+: 3.8 (2.2 - 6.5) -: 0.6 (0.4 - 0.9)	18.8%	96.5%	0.676
≥5	28.5% (11 - 52)	96.2% (93 - 97)	+: 7.6 (3.2 - 17.9) -: 0.7 (0.6 - 0.9)	31.5%	95.7%	0.624

AUC: Area under the curve, 95% CI: 95% confidence interval, LHR: Likelihood ratio, PPV: Positive predictive value, NPV: Negative predictive value

The analysis considering all patients with a positive result in the uric acid parameter on the scale showed that the best cutoff point for considering the test as positive was 4, with exactly the same values (sensitivity, specificity, LHR +/- and AUC) as for the original scale without uric acid (Appendix 3).

### Comparison of best cut-off points: fullPIERS vs EFRCP

**Figure 4:**
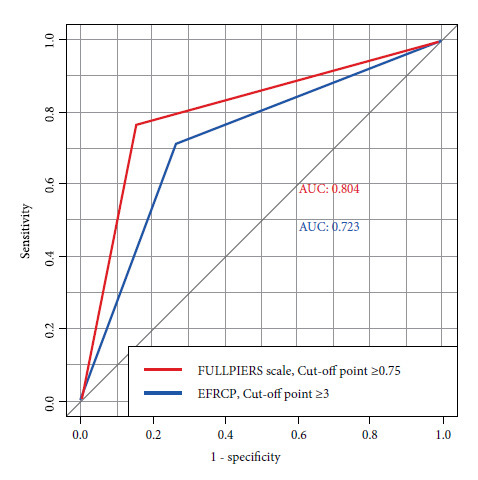
FullPIERS scale VS EFRCP: Comparison of best cut-off points.

No significant difference was reported between the two when comparing the best cut-off points of both scales using the AUC of each, with a p=0.215 according to the Delong Test (Figure 4).

Red line: ROC curve of the FULLPIERS Scale with a cut-off point at 0.75. Blue line: ROC curve of the Risk Factor Scale for Preeclampsia Complications with a cut-off point at 3.

## DISCUSSION

In this study, we did not find any statistically significant differences between the EFRCP and fullPIERS scales in their ability to predict complications of preeclampsia in our population. This finding suggests that both scales have similar diagnostic performance in our context, implying that either could be used in clinical practice without strict preference, depending on the characteristics and resources of the care setting.

This study evaluated the performance of the fullPIERS and EFRCP scales for predicting complications in preeclampsia in a population of 367 pregnant women, of whom 21 had serious complications, the most common being obstetric hemorrhage requiring surgical intervention or transfusion and difficult-to-control hypertension. The prevalence of preeclampsia complications in our sample was 6%, lower than the 10% reported in the literature, which could be explained by access to health services and increased prenatal care in the early stages of pregnancy ^20^.

When evaluating the EFRCP scale, we found that the best cutoff point was 3, with a sensitivity of 71%, specificity of 73%, and an AUC of 0.7227. This cutoff point was lower than the value of 4 proposed by the original study ^10^, which may be attributed to the lack of uric acid measurement in our institution. However, additional analyses suggest that the absence of this parameter did not significantly alter the results in terms of sensitivity and specificity, although it did affect the determination of the most appropriate cutoff point.

The best cut-off point was 0.75% for the full PIERS scale, with a sensitivity of 76%, specificity of 84%, and an AUC of 0.804, lower than the 30% suggested in the original study. Different studies in other countries, such as Canada, the Netherlands, Mexico, and Brazil ^21^^-^^23^, have shown significant variability in the optimal cutoff points for this scale, which could reflect population or contextual differences that affect its performance. The heterogeneity in optimal cut-off points reported by other studies and by our study could suggest that the performance of fullPIERS is sensitive to the specific characteristics of each population.

This study evaluated two scales for predicting complications of preeclampsia, as these models are a simple and optimal way to assess patients diagnosed with preeclampsia in the office, emergency room, or hospital, in order to classify them and provide them with appropriate management and follow-up. The analysis of these scales provides clinicians and the scientific community with an overview of how these scales work in our setting. Thus, we have different cut-off points on both scales with optimal AUC values, demonstrating their correct performance as a clinical tool. This aspect is important because, depending on the cut-off point, each tool has a different sensitivity and specificity, which could support the future use of these scales in the clinical setting as tools for ruling out or detecting preeclampsia in a timely manner, as required by healthcare personnel during their practice.

The study is supported by a representative sample from a public hospital and a detailed analysis of the cut-off points and performance of both scales. This study represents the reality of a public hospital, which has similar characteristics to other establishments in the country and other contexts in Latin America. Our findings provide a critical view of its applicability in our context. However, the retrospective design implies the possibility of incomplete medical records, although only six cases with missing data were identified. Another limitation was the lack of uric acid measurement, an obstacle that was partially overcome through complementary analyses suggesting that this deficiency did not significantly affect the results.

In conclusion, both scales performed optimally in predicting complications in pregnant women with preeclampsia. The Preeclampsia Risk Factors Scale showed the best results when evaluated with a cutoff point of 3, while the fullPIERS scale demonstrated the best performance with a cutoff point of 0.75%. When compared at their best cut-off points, the two scales did not show any statistical differences, leaving the choice of scale to the treating physician based on their needs and available resources. Given our results, we recommend that each scale be evaluated in the setting where it is to be used, due to the evidence of differences in the cut-off points obtained compared to the original studies.
