# Supplementary material for: Assessment of the ability to predict complications of the Risk Factor Scale for Pre-eclampsia Complications and the fullPIERS scale in pregnant women in a Hospital in Lima, Peru
Source: Rev Peru Med Exp Salud Publica. 2025 Feb 19;42(1):46–53. doi: 10.17843/rpmesp.2025.421.14041 (PMC12176016; doi:10.17843/rpmesp.2025.421.14041)
Supplement: Supplementary material. — Available in the electronic version of the RPMESP. [file rpmesp-42-01-14041-s001.docx]

**MATERIAL SUPLEMENTARIO**

**Anexo 1.** Variables de la escala fullPIERS

| **Variable** | **Definición** | **Valor del registro** |
| --- | --- | --- |
| Edad gestacional al momento del diagnóstico de preeclampsia | Semanas de embarazo obtenidas por FUR o ecografía del primer trimestre hasta el diagnóstico de preeclampsia. | Valor numérico (semanas) |
| Disnea | Sensación de falta de aire o también manifestada cómo dificultad para respirar. | Presente  Ausente |
| Recuento de plaquetas | Número total plaquetario obtenido de una muestra de sangre | Valor numérico (mm3) |
| Creatinina sérica | Metabolito obtenido de la degradación de las proteínas. | Valor numérico (mg/dL) |
| TGO | Enzima localizada en hígado, músculo esquelético, cerebro, glóbulos rojos, miocardio, entre otros. Su aumento se asocia a daño tisular. | Valor numérico (Ul/l) |
| Saturación de Oxígeno | Medición porcentual del nivel de saturación de oxígeno en la hemoglobina. Tomado por medio de un pulsioxímetro. | Valor numérico(%) |

**Anexo 2**. Variables de la Escala de Factores de Riesgo para Complicaciones de Preeclampsia

| **Variable** | **Definición** | **Valor del registro** |
| --- | --- | --- |
| Edad | Años desde el nacimiento hasta el día que ingresó al estudio. El punto de corte para considerarlo cómo positivo para la escala es de ≤ 20 años. | Valor numérico (años) |
| Cefalea | Sensación de dolor de cabeza | Sí  No |
| Oliguria | Cuadro caracterizado por diuresis menor a 20-30 mL/h durante 2 horas. El punto de corte para considerarlo cómo positivo para la escala es de ≤ 0.5 ml/kg/d. | Presente  Ausente |
| Deshidrogenasa láctica (DHL) | Enzima que se encuentra en diversos tejidos (miocardio, cerebro, riñones, glóbulos rojos, hígado, entre otros) su elevación se asocia con daño o necrosis celular. El punto de corte para considerarlo cómo positivo para la escala es de ≥400 UI/L. | Valor numérico (UI/L) |
| Índice normalizado internacional de tiempo de protrombina (INR) | Examen laboratorial destinado a la medición de la vía extrínseca de la coagulación. El punto de corte para considerarlo cómo positivo para la escala es de ≥1.2. | Valor numérico |
| Volumen plaquetario medio | Medida que se obtiene de la curva de distribución de los tamaños plaquetarios. El punto de corte para considerarlo cómo positivo para la escala es de ≥8.5 fL. | Valor numérico (fL) |
| Ácido úrico sérico | Metabolito que se obtiene de la degradación de las purinas, sus valores disminuyen aproximadamente en 30% durante la gestación. El punto de corte para considerarlo cómo positivo para la escala es de ≥6 mg/dl. | Valor numérico (mg/dL) |
| Edad gestacional al momento del diagnóstico de preeclampsia | Semanas de embarazo obtenidas por FUR o ecografía del primer trimestre hasta el diagnóstico de preeclampsia. | Valor numérico(semanas) |
| Disnea | Sensación de falta de aire o también manifestada cómo dificultad para respirar. | Presente  Ausente |
| Recuento de plaquetas | Número total plaquetario obtenido de una muestra de sangre. El punto de corte para considerarlo cómo positivo para la escala es de ≥150 000 mm^3^. | Valor numérico (mm3) |
| Creatinina sérica | Metabolito obtenido de la degradación de las proteínas. El punto de corte para considerarlo cómo positivo para la escala es de ≥0.9 mg/dL. | Valor numérico (mg/dL) |
| TGO | Enzima localizada en hígado, músculo esquelético, cerebro, glóbulos rojos, miocardio, entre otros. Su aumento se asocia a daño tisular. El punto de corte para considerarlo cómo positivo para la escala es de ≥40 UI/L. | Valor numérico (Ul/L) |

| **Anexo 3.** Puntos de corte y parámetros evaluados en la Escala de Factores de Riesgo de complicaciones de preeclampsia con ácido úrico |
| --- |

| **Punto de corte** | **Sensibilidad** | **Especificidad** | **LHR** | **VPP** | **VPN** | **AUC** |
| --- | --- | --- | --- | --- | --- | --- |
| **≥2** | 100% (83 – 100%) | 0.3% (0 - 1.6%) | **+:** 1.0(0.9 - 1.0)  **-:** 0.0 | 5.7% | 100% | 0.501 |
| **≥ 3** | 76.2% (52 – 91%) | 40.5% (35 – 45%) | **+:** 1.3 (1.0 - 1.6)  **-:** 0.6 (0.3 - 1.3) | 7.2% | 96.5% | 0.583 |
| **≥ 4** | 71.4% (47 – 88%) | 40.5% (35 – 45%) | **+:** 2.6 (1.9 - 3.7)  **-:** 0.4 (0.2 - 0.8) | 13.8% | 97.7% | 0.723 |
| **≥ 5** | 47.6% (25 – 70%) | 87.6% (83 – 90%) | **+:** 3.8 (2.3 - 6.5)  **-:** 0.6 (0.4 - 0.9) | 18.9% | 96.5% | 0.676 |
| **≥ 6** | 28.6% (11 – 52%) | 96.2% (93 – 97%) | **+:** 7.6 (3.2 - 17.9)  **-:** 0.7 (0.6 - 0.9) | 31.6% | 95.7% | 0.624 |

AUC: Área bajo la curva, IC95%: Intervalo de Confianza al 95%, LHR: Likelihood Ratio, VPP: Valor predictivo positivo, VPN: Valor predictivo negativo
