# Supplementary material for: Assessment of the ability to predict complications of the Risk Factor Scale for Pre-eclampsia Complications and the fullPIERS scale in pregnant women in a Hospital in Lima, Peru
Source: Rev Peru Med Exp Salud Publica. 2025 Feb 19;42(1):46–53. doi: 10.17843/rpmesp.2025.421.14041 (PMC12176016; doi:10.17843/rpmesp.2025.421.14041)
Supplement: Translation

# Translation: Evaluación de la capacidad para predecir complicaciones de la Escala de Factores de Riesgo para Complicaciones de Preeclampsia y la escala fullPIERS en gestantes de un Hospital de Lima, Perú

**Authors:** Patricia N. Aquino-Vásquez, Luis GM. Chuquipoma-Zanabria, Maria Lazo-Porras, Mónica Flores-Noriega

**Affiliations:** 1 Escuela de Medicina, Universidad Peruana Cayetano Heredia, Lima, Perú.; 2 CRONICAS, Centro de Excelencia en Enfermedades Crónicas, Universidad Peruana Cayetano Heredia, Lima, Perú.; 3 Clínica Angloamericana. Lima, Perú.; 4 Hospital Cayetano Heredia, Lima, Perú.

**Keywords:** Preeclampsia, Complicaciones del Embarazo, Gestación

## Abstract

**Objetivos.:**

Evaluar las capacidades para predecir complicaciones de preeclampsia de la Escala de Factores de Riesgo para Complicaciones de Preeclampsia (EFRCP) y la escala fullPIERS (*Pre-eclampsia Integrated Estimate of RiSk*) en gestantes con diagnóstico de preeclampsia que ingresan al servicio de gineco-obstetricia de un hospital de referencia, desde octubre del 2021 a diciembre del 2022.

**Materiales y métodos.:**

Estudio de diseño de cohorte retrospectivo, se recolectaron los datos de historias clínicas de pacientes diagnosticadas con preeclampsia y se aplicó ambas escalas (EFRCP y fullPIERS). Con estos resultados se halló la sensibilidad, especificidad y el área bajo la curva ROC (AUC) tomando diferentes puntos de corte. Se seleccionó como mejor puntaje aquel con una mayor AUC. Se exploró las diferencias entre las escalas comparando sus AUC.

**Resultados.:**

Se incluyeron a 367 gestantes. La EFRCP presentó 71% de sensibilidad, 73% de especificidad y AUC de 0,722 con un punto de corte de 3 puntos. Mientras que en la escala de fullPIERS obtuvimos 76%, 84% y 0,804 respectivamente con el punto de corte de 0,75%.

**Conclusiones.:**

Ambas escalas pueden ser de utilidad para identificar gestantes en riesgo de complicaciones con puntos de corte diferentes a los definidos internacionalmente.

## INTRODUCCIÓN

La preeclampsia es una enfermedad multisistémica progresiva incluida dentro de los desórdenes hipertensivos del embarazo. Fisiopatológicamente, la preeclampsia se desarrolla en dos etapas. La primera etapa se caracteriza por anomalías en la vascularización uterina en el primer trimestre, lo que lleva a una placentación anómala e isquemia placentaria. Es en esta etapa donde se relaciona a los factores angiogénicos, que son proteínas clave en el proceso de formación de nuevos vasos sanguíneos, fundamental para el crecimiento, desarrollo y reparación de tejidos. Entre los más relevantes se encuentran el factor de crecimiento endotelial vascular, que promueve la proliferación y migración de las células endoteliales, y el factor de crecimiento derivado de plaquetas, que juega un papel crucial en la estabilización de los vasos recién formados. La liberación de estos factores provoca una disfunción endotelial sistémica, marcando el inicio de la segunda etapa, que se manifiesta con daño vascular agudo y/o hipoperfusión ^1^. 

Según el Colegio Americano de Obstetras y Ginecólogos (ACOG) el diagnóstico de preeclampsia se establece con hipertensión arterial (HTA) de reciente aparición pasadas las 20 semanas de gestación conjuntamente con proteinuria. Sin embargo, existen otras manifestaciones clínicas tales cómo trombocitopenia, insuficiencia renal, edema pulmonar, hepatopatía, alteraciones visuales o dolor en hipocondrio derecho cuya existencia de manera conjunta con la HTA es suficiente para establecer el diagnóstico de preeclampsia con criterios de severidad ^2^^,^^3^. Los trastornos hipertensivos del embarazo afectan al 10% de las gestaciones a nivel mundial. En países desarrollados, las complicaciones de anestesia y cesáreas son la principal causa de muerte gestacional, mientras que, en América Latina, la preeclampsia es la causa principal, representando el 25,7% de las muertes en 2011. La incidencia de preeclampsia severa varía del 2-5% en países desarrollados y hasta el 18% en países en desarrollo ^4^^,^^5^.

Debido a las graves consecuencias de la preeclampsia en la salud materno-fetal, es crucial tener una escala con parámetros confiables para predecir tempranamente sus complicaciones, que incluyen muerte materna, eclampsia, eventos cerebrales agudos, tromboembolismo pulmonar, hematoma hepático, lesión renal aguda, coagulopatías y trombocitopenia severa ^6^^-^^8^. Se han realizado estudios, como el ensayo PETRA ^9^, para predecir resultados adversos en embarazadas con preeclampsia. Este ensayo comparó estrategias de manejo en casos de preeclampsia severa temprana y su impacto en madre y feto, lo que permitió desarrollar escalas para predecir complicaciones severas de esta enfermedad. La escala fullPIERS ^10^^,^^11^, desarrollada entre 2003 y 2010, es un modelo de predicción de riesgo para la preeclampsia, basado en datos de más de 2000 mujeres. Tiene una sensibilidad del 76% y una especificidad del 87% para predecir desenlaces maternos adversos dentro de las 48 horas de diagnóstico. Evalúa 6 criterios y puede predecir complicaciones hasta 7 días antes. Aunque se busca su implementación global, su desarrollo en países de altos ingresos podría afectar su aplicabilidad en Latinoamérica.

Se tiene otra escala construida en México por Elizalde y colaboradores en 2014, es la Escala de Factores de Riesgo para Complicaciones de Preeclampsia (EFRCP), se elaboró a partir de un estudio de casos y controles en pacientes con preeclampsia que cursaron o no con complicaciones ^12^, se obtuvo factores de riesgo capaces de formar parte de un instrumento que predice las complicaciones de preeclampsia, la escala cuenta con 12 parámetros y presenta una sensibilidad de 93% y especificidad del 80% ^13^. Ha sido aplicada exclusivamente en el país en la que fue diseñada, México, sin estudios en otros países que la hayan evaluado a la fecha. 

Las escalas EFRCP y fullPIERS pueden ser útiles en la toma de decisiones para el tratamiento temprano de la preeclampsia, con el objetivo de reducir las consecuencias negativas tanto para la madre como para el feto. Dado que la preeclampsia es una de las principales causas de mortalidad materno-fetal en países en desarrollo, el uso de estas escalas se justifica para identificar a las mujeres embarazadas con mayor riesgo de complicaciones y brindar atención médica oportuna. Esta investigación tiene como objetivo evaluar la capacidad predictora de complicaciones de la preeclampsia utilizando las escalas EFRCP y fullPIERS en pacientes diagnosticadas con preeclampsia en un hospital de Lima durante el período de octubre de 2021 a diciembre de 2022.

**MENSAJE CLAVE**

Motivación para realizar el estudio. La preeclampsia es una causa significativa de complicaciones maternas y fetales, en Perú no está estandarizado el uso de alguna herramienta que mejore la identificación temprana y que optimice el tratamiento de gestantes con preeclampsia.

Principales hallazgos. Ambas escalas predijeron eficazmente las complicaciones de la preeclampsia, en sus puntos de corte óptimos.

Implicancias. Las escalas predictivas evaluadas aportan evidencia para futuras investigaciones esenciales para reducir las complicaciones y sus repercusiones materno- fetales asociadas a la preeclampsia.

## MATERIALES Y MÉTODOS

### Diseño del estudio y contexto

Estudio de cohorte retrospectivo. Desarrollado en el Hospital Cayetano Heredia el cuál corresponde a un hospital de nivel de complejidad III-1, se encuentra ubicado al norte de Lima, Perú. El área de influencia correspondiente a este hospital tiene una población de 3 143 582 habitantes, los cuales generalmente son de recursos económicos limitados y se encuentran afiliados al Sistema Integral de Salud (SIS) ^14^^,^^15^^)^ Además según reportes del servicio de Obstetricia en el 2019 tuvieron un número de partos anuales de casi 5000.

### Población

Gestantes con el diagnóstico de preeclampsia que ingresaron al servicio de Obstetricia del Hospital Cayetano Heredia desde el mes de octubre del 2021 al mes de diciembre del año 2022. Las pacientes incluidas en el estudio son gestantes a partir de la semana 20 de gestación con los criterios para el diagnóstico de preeclampsia ^16^: Presión alta determinada por presión sistólica ≥140 y/o presión diastólica ≥90 en dos ocasiones con una diferencia de al menos 4 horas después de la semana 20 de gestación en una mujer con una presión previa normal, presión sistólica ≥160 y/o presión diastólica ≥110 mm Hg (se considera hipertensión severa) proteinuria: ≥300 mg en orina de 24 horas, índice proteína/creatinina ≥0,3 mg/dl o lectura de tira reactiva 2+. En caso no haya proteinuria, se consideró el diagnóstico cuando la paciente manifieste hipertensión gestacional de nuevo comienzo y presente alguno de los siguientes criterios: Trombocitopenia (conteo de plaquetas ≤100 000 x 10 *9/L), insuficiencia renal (creatinina sérica >1,1 mg/dL o que doble el valor sérico en ausencia de enfermedad renal), alteración de función hepática (elevación de transaminasas dos veces de la concentración normal), dolor severo persistente en el cuadrante superior derecho o dolor epigástrico sin una alternativa diagnóstica, edema pulmonar, dolor de cabeza que no responde a la medicación y no se explica por un diagnóstico alternativo, síntomas visuales (visión borrosa, luces, escotomas, chispas).

Las pacientes excluidas del estudio fueron las que cuentan con diagnóstico de preeclampsia que adicionalmente presentaron los siguientes diagnósticos: Muerte materna, eclampsia, evento cerebro vascular, edema pulmonar, tromboembolia pulmonar, síndrome de HELLP, hematoma hepático, lesión renal aguda AKI II o III, diálisis, trombocitopenia severa, coagulopatía, hemorragia obstétrica, hipertensión arterial refractaria. 

### Muestra

Para determinar el tamaño de la muestra se empleó una calculadora virtual ^17^^,^^18^. Se utilizaron los valores de sensibilidad y especificidad de la escala fullPIERS que corresponden a 76% y 87% respectivamente ^8^. Se utilizó como prevalencia de las complicaciones el valor de 20% que corresponde a la prevalencia de la eclampsia reportada en un estudio previo ^19^. Con una precisión de 10% y un nivel de confianza del 95%. El tamaño muestral obtenido fue de 351 pacientes.

### Definición operacional de variables

En este estudio la variable desenlace son las complicaciones maternas de la preeclampsia, mientras que las variables predictoras son los resultados de la escala fullPIERS y los resultados de la EFRCP. 

Las definiciones y mediciones de cada una se explican a continuación: Las complicaciones de la preeclampsia son complicaciones derivadas de la enfermedad hipertensiva del embarazo o preeclampsia. La información fue obtenida de la historia clínica, en el estudio, el valor “sí” corresponde a presentar al menos una de las siguientes complicaciones: coagulopatía, eclampsia, edema pulmonar, hemorragia obstétrica que requiere intervención quirúrgica o hemoderivados, hipertensión de difícil control, lesión renal aguda, síndrome de HELLP o muerte materna. 

La Escala fullPIERS es una herramienta diseñada para la predicción de la aparición de complicaciones de la preeclampsia dentro de las 48 horas subsecuentes a la hospitalización en una paciente con el diagnóstico de preeclampsia, sin embargo, la escala demostró resultados favorables en predecir complicaciones incluso dentro de los primeros 7 días posteriores a la hospitalización (AUC >0,7). Las variables que considera son: edad gestacional, dolor torácico o disnea, saturación de oxígeno, conteo de plaquetas, creatinina sérica y aspartato aminotransferasa sérica (AST) tomadas al momento de la hospitalización de la paciente; dichos valores son registrados en la calculadora oficial de la escala ^9^ cuyo resultado fue expresado en porcentajes indicando la probabilidad del desarrollo de una o más complicaciones de preeclampsia. El punto de corte determinado para considerar a una paciente como de alto riesgo para el desarrollo de uno o más complicaciones de preeclampsia es de ≥30% obteniendo una sensibilidad de 75,5%, especificidad 86,9%, y un área bajo la curva ROC (AUC) de 0,88. Se detallan los conceptos pertinentes de cada una de las variables de la Escala FullPIERS en el Material Suplementario Anexo 1.

La Escala de Factores de Riesgo para Complicaciones de Preeclampsia (EFRCP) es una herramienta que permite la predicción de complicación de la preeclampsia, es una escala que se construyó y validó en México ^12^. Durante el desarrollo de la escala, se determinaron 12 parámetros. Éstos son: edad materna, edad gestacional al diagnóstico de preeclampsia, cefalea intensa, disnea, plaquetas, volumen plaquetario medio, INR, creatinina sérica, ácido úrico sérico, transaminasa glutámica oxalacética y deshidrogenasa láctica sérica, a cada una de las variables se le asignó un puntaje; finalmente la sumatoria total del puntaje de las variables positivas determinó el puntaje final de la escala. Fue así que se asignó una acción según el puntaje de la escala obtenido, 0 puntos descartan complicación, 1-2 puntos se debe hacer seguimiento por parte del médico y hay baja probabilidad de complicaciones, 3 puntos significan que la paciente debe tener evaluación y seguimiento por personal de terapia intensiva sin necesidad de ingresar a unidad de cuidados intensivos, por último 4 puntos a más significa un alto riesgo de complicación y se sugiere el ingreso a unidad de cuidados intensivos; este punto de corte presentó los siguientes valores estadísticos: sensibilidad: 93%, especificidad: 80% y AUC: 0,98. Se detallan los conceptos pertinentes de cada una de las variables de la escala EFRCP en el Anexo 2.

Se incluyeron variables sociodemográficas para describir las características de la población. Estas corresponden a edad materna (<18, 18-34, ≥35), país de origen (Perú, Venezuela, Colombia), edad gestacional (≤27, 28-36, ≥37), número de gestaciones (1, ≥2), paridad (0, ≥1) y sexo fetal (masculino, femenino). 

### Procedimientos y técnicas

Se revisó cada historia clínica semanalmente de las pacientes gestantes hospitalizadas de emergencia y se identificó a aquellas que cumplan los criterios de inclusión y exclusión por conveniencia. Se construyó una ficha de recolección de datos, cuyo contenido fue llenado con los datos clínicos y demográficos de las pacientes, criterios diagnósticos de la preeclampsia, datos para verificar las complicaciones con sus respectivos criterios diagnósticos, datos de las variables predictoras de la calculadora fullPIERS, datos de las variables de la EFRCP, además del dato de la fecha de aplicación del formulario, hospitalización, alta, fallecimiento, parto y diagnóstico de las complicaciones. Para la recolección de datos, la ficha fue llenada y almacenada en la plataforma virtual de Google Forms, cuyo acceso estuvo habilitado únicamente para los investigadores.

### Análisis estadístico

Inicialmente, se describieron las variables sociodemográficas y obstétricas importantes, reportando las variables categóricas en frecuencias y porcentajes. Se analizó la base de datos utilizando RStudio, se realizó un análisis individual de cada escala y se buscó determinar cuál fue el mejor punto de corte para catalogar a una paciente como de alto riesgo de complicaciónes; para esto cada escala fue examinada con distintos puntos de corte, para la elección de estos puntos de corte lo primero que se hizo fue hallar el punto de corte con la mejor AUC; una vez hallada, se eligieron los demás puntos en base a la proximidad al punto de corte del estudio original y de nuestro estudio. En el caso de la escala fullPIERS, se tomaron en cuenta como puntos de corte los siguientes valores: 30%, 10%, 5%, 1%, 0,75% y 0,5% y en el caso de la escala EFRCP se tomaron como puntos de corte los valores ≥1, ≥2, ≥3, ≥4 y ≥5.

Para evaluar el rendimiento diagnóstico de las escalas, se usó como estándar de oro el diagnóstico de complicaciones de la preeclampsia. Se calculó la sensibilidad, especificidad, valor predictivo positivo (VPP), valor predictivo negativo (VPN), razón de probabilidades positivas (LHR+) y negativas (LHR-), y el AUC. Se evaluó el rendimiento diagnóstico considerando la presencia o ausencia de complicaciones. Para hallar el mejor punto de corte se seleccionó el punto o valor de mayor sensibilidad y especificidad dado por el AUC.

Se comparó el rendimiento diagnóstico de la calculadora fullPIERS y la escala EFRCP utilizando el AUC y un test de proporciones (Test de DeLong). Ante la falta del parámetro "ácido úrico" en la escala EFRCP, se propuso un análisis paralelo incluyendo dicho parámetro cómo positivo para todos y se exploraron las diferencias con el estudio sin ácido úrico; posteriormente se eligió al análisis con el mejor rendimiento diagnóstico para que sea comparado con la escala fullPIERS.

### Aspectos éticos

Se tuvo aprobaciones de los comités de ética de la Universidad Peruana Cayetano Heredia y del Hospital Cayetano Heredia para asegurar que el proyecto cumpla con las normas de Buenas Prácticas de Investigación. 

## RESULTADOS

Se revisaron 400 historias clínicas de las cuales 28 se descartaron por datos erróneos. Se registraron 373 historias en la base de datos, 6 de ellas presentaban datos incompletos, por lo que finalmente 367 historias fueron registradas en la base de datos oficial la cual fue exportada a formato de Excel (Figura 1). 

**Figura 1:** Flujograma de selección de participantes.

### Características de la población de estudio

El 1,9% de las gestantes en la muestra fueron adolescentes y el 25,6% tuvieron edad materna avanzada. El 22,6% fueron de procedencia extranjera. (Tabla 1). El 6% (21 participantes) de las pacientes presentaron alguna complicación de preeclampsia. Las dos complicaciones más comunes encontradas fueron la hemorragia obstétrica que requirió intervención quirúrgica o hemoderivados e hipertensión de difícil control con 5 casos en cada uno.

**Tabla 1:** Análisis descriptivo de la población incluida.

Características		n (%)
Edad materna		
	<18	7 (1,9)
	18 - 34	266 (72,5)
	≥ 35	94 (25,6)
País de origen		
	Perú	284 (77,4)
	Venezuela	82 (22,3)
	Colombia	1 (0,3)
Edad gestacional		
	≥ 37	261 (71,1)
	36-28	103 (28,1)
	≤27	3 (0,8)
Número de gestaciones		
	1	107 (29,2)
	≥2	260 (70,8)
Paridad		
	0	141 (38,4)
	≥1	226 (61,6)
Sexo fetal		
	Masculino	194 (52,9)
	Femenino	173 (47,1)
Complicaciones		
	Sí	21 (5,7)
	No	346 (94,3)
Tipo de complicaciones		
	Coagulopatía	2 (0,5)
	Eclampsia	2 (0,5)
	Edema pulmonar	1 (0,3)
	Hemorragia obstétrica que requiere intervención quirúrgica o hemoderivados	5 (1,3)
	Hipertensión de difícil control	5 (1,3)
	Lesión renal aguda	3 (0,8)
	Síndrome de HELLP	2 (0,5)
	Muerte materna	1 (0,3)

### Análisis de escala fullPIERS

Se llegó a la conclusión de determinar como mejor punto de corte al valor de 0,75% obteniéndose así los siguientes resultados: Sensibilidad 76%, especificidad 84%, LHR + 4,9 LHR - 0,3, VPP 23%, VPN 98%, AUC 0,804 (Figura 2). Los resultados de los diferentes puntos de corte se encuentran en la Tabla 2.

**Figura 2:** Escala fullPIERS: Comparativa de puntos de corte.

**Tabla 2:** Puntos de corte y parámetros evaluados en la escala fullPIERS.

Punto de corte	Sensibilidad (IC 95%)	Especificidad (IC 95%)	LHR (IC 95%)	VPP	VPN	AUC
≥ 30	4,8% (0 - 23)	99,7% (98 - 99)	+: 16,5 (1,1 - 254,2) -: 0,9 (0,86 - 1,05)	50,0%	94,5%	0,522
≥ 10	14,3% (0,3 - 36)	99,4% (97 - 99)	+: 24,7 (4,4 - 139,9) -: 0,9 (0,7 - 1,0)	60,0%	95,0%	0,568
≥ 5	19,1% (0,5 - 41)	99,1% (97 - 99)	+: 22,0 (5,2 - 91,8) -: 0,8 (0,6 - 1,0)	57,1%	95,3%	0,591
≥ 1	61,9% (38 - 81)	89,9% (86 - 92)	+: 6,1 (3,8 - 9,7) -: 0,4 (0,2 - 0,7)	27,1%	97,5%	0,759
≥ 0,75	76,2% (52 - 91)	84,6% (80 - 88)	+: 5,0 (3,5 - 7,0) -: 0,3 (0,1 - 0,6)	23,2%	98,3%	0,804
≥ 0,5	85,7% (63 - 96)	68,2% (63 - 73)	+: 2,7 (2,1 - 3,4) -: 0,2 (0,1 - 0,6)	14,1%	98,7%	0,769

AUC: Área bajo la curva, IC95%: Intervalo de confianza al 95%, LHR: Likelihood ratio, VPP: Valor predictivo positivo, VPN: Valor predictivo negativo

### Análisis de Escala de Factores de Riesgo para Complicaciones de Preeclampsia

Realizado el análisis se determinó al punto de corte 3 como el mejor valor para considerar al paciente como positivo según la escala; obteniéndose así los siguientes resultados: Sensibilidad 71%, especificidad 73%, LHR+ 2,6, LHR- 0,3 AUC 0,722 (Tabla 3 y Figura 3).

**Tabla 3:** Puntos de corte y parámetros evaluados en la Escala de Factores de Riesgo de complicaciones de preeclampsia sin ácido úrico.

Punto de corte	Sensibilidad (IC 95%)	Especificidad (IC 95%)	LHR (IC 95%)	VPP	VPN	AUC
≥1	100% (84 - 100)	0% (0 - 1)	+: 1,0 (0,9 - 1,0) -: 0,0	5,7%	100%	0,501
≥ 2	76,2% (52 - 91)	40,4% (35 - 45)	+: 1,3 (0,9 - 1,6) -: 0,6 (0,3 - 0,3)	7,2%	96,5%	0,583
≥3	71,4% (47 - 88)	73,1% (68 - 78)	+: 2,7 (1,9 - 3,6) -: 0,4 (0,1 - 0,7)	13,8%	96,7%	0,723
≥ 4	47,6% (25 - 70)	87,6% (83 - 90)	+: 3,8 (2,2 - 6,5) -: 0,6 (0,4 - 0,9)	18,8%	96,5%	0,676
≥5	28,5% (11 - 52)	96,2% (93 - 97)	+: 7,6 (3,2 - 17,9) -: 0,7 0,6 - 0,9)	31,5%	95,7%	0,624

AUC: Área bajo la curva, IC95%: Intervalo de confianza al 95%, LHR: Likelihood ratio, VPP: Valor predictivo positivo, VPN: Valor predictivo negativo

**Figura 3:** Escala EFRCP sin ácido úrico: Comparativa de puntos de corte.

En el análisis considerando a todas las pacientes con un resultado positivo en el parámetro de ácido úrico en la escala; se obtuvo que el mejor punto de corte para considerar la prueba como positiva fue de 4 con exactamente los mismos valores (sensibilidad, especificidad, LHR +/- y AUC) que para la escala original sin ácido úrico (Anexo 3). 

### Comparación de mejores puntos de corte: fullPIERS vs EFRCP

En la comparación entre los mejores puntos de corte de ambas escalas mediante el AUC de cada una de ellas, se reportó que no existe diferencia significativa entre ambas, con un p=0,215 según el Test de Delong (Figura 4).

**Figura 4:** Escala fullPIERS VS EFRCP: Comparativa de mejores puntos de corte.

Línea roja: Curva ROC de la Escala fullPIERS con punto de corte en 0,75. Línea azul: Curva ROC de la Escala de Factores de Riesgo para Complicaciones de Preeclampsia con punto de corte en 3

## DISCUSIÓN

En este estudio, no se encontraron diferencias estadísticamente significativas entre las escalas EFRCP y fullPIERS en su capacidad para predecir complicaciones en preeclampsia en nuestra población. Este hallazgo sugiere que ambas escalas tienen un desempeño diagnóstico similar en nuestro contexto, lo cual implica que cualquiera de las dos podría ser utilizada en la práctica clínica sin una preferencia estricta, dependiendo de las características y recursos del entorno de atención. 

La presente investigación evaluó el rendimiento de las escalas de predicción de complicaciones en preeclampsia fullPIERS y EFRCP en una población de 367 gestantes, de las cuales 21 presentaron complicaciones graves, siendo las más frecuentes la hemorragia obstétrica que requirió intervención quirúrgica o transfusión y la hipertensión de difícil control. La prevalencia de complicaciones de preeclampsia en nuestra muestra fue del 6%, inferior al 10% reportado en la literatura, lo cual podría explicarse por el acceso a los servicios de salud y el incremento en los controles prenatales en etapas tempranas del embarazo ^20^.

Al evaluar la escala EFRCP, encontramos que el mejor punto de corte fue 3, con una sensibilidad del 71%, especificidad del 73% y un AUC de 0.7227. Este punto de corte es menor al valor de 4 propuesto por el estudio original ^10^, lo cual puede atribuirse a la falta de medición de ácido úrico en nuestra institución. No obstante, los análisis adicionales sugieren que la ausencia de este parámetro no alteró significativamente los resultados en términos de sensibilidad y especificidad, aunque sí en la determinación del punto de corte más adecuado.

Para la escala fullPIERS, el mejor punto de corte fue de 0,75%, con una sensibilidad del 76%, especificidad del 84% y un AUC de 0,804, menor que el 30% sugerido en el estudio original. Diferentes estudios en otros países, como Canadá, Países Bajos, México y Brasil ^21^^-^^23^, han mostrado una variabilidad significativa en los puntos de corte óptimos para esta escala, lo cual podría reflejar diferencias poblacionales o contextuales que afectan su rendimiento. La heterogeneidad en los puntos de corte óptimos observada en otros estudios y en el presente podría sugerir que el desempeño de fullPIERS es sensible a las características específicas de cada población.

En este estudio se evaluaron dos escalas predictoras de complicaciones de preeclampsia, ya que, estos modelos son una manera sencilla y óptima de evaluar a las pacientes que son diagnosticadas con preeclampsia en consultorio, emergencia u hospitalización para clasificarlas y proporcionarles un adecuado manejo y seguimiento. El análisis de estas escalas permite brindar al clínico y a la sociedad científica un panorama del funcionamiento de dichas escalas en nuestro medio; es así que tenemos diversos puntos de corte en ambas escalas que cuentan con AUC de valores óptimos demostrando así su correcto desempeño como una herramienta clínica; este aspecto es importante ya que dependiendo del punto de corte cada herramienta cuenta con una sensibilidad y especificidad distinta lo que podría apoyar en un futuro al empleo de estas escalas en el ámbito clínico cómo herramientas de descarte o de detección oportuna según el personal sanitario lo requiera en su práctica. 

El estudio cuenta con el respaldo de una muestra representativa de un hospital público y de un análisis detallado de los puntos de corte y rendimiento de ambas escalas. Este estudio representa la realidad de un hospital público, que tiene características similares a otros establecimientos del país y de otros contextos de Latinoamérica, los hallazgos aportan una visión crítica sobre su aplicabilidad en nuestro contexto. Sin embargo, el diseño retrospectivo implica la posibilidad de registros incompletos en las historias clínicas, aunque solo se identificaron seis casos con datos faltantes. Otra limitación fue la falta de medición del ácido úrico, un obstáculo superado en parte mediante análisis complementarios que sugieren que esta carencia no afectó de manera significativa los resultados.

En conclusión, ambas escalas evaluadas durante el estudio presentaron desempeños óptimos en la predicción de complicaciones en gestantes con preeclampsia. La Escala de factores de riesgo para complicaciones de Preeclampsia mostró los mejores resultados al ser evaluada con el punto de corte de 3, mientras que la escala fullPIERS, demostró tener el mejor desempeño al tener como punto de corte el valor de 0,75%. Ambas escalas al ser comparadas en sus mejores puntos de corte no mostraron ser diferentes estadísticamente lo que deja a criterio del médico tratante la elección de cada escala según sus necesidades y recursos disponibles. Dados estos resultados, recomendamos se evalúe cada escala en el propio medio en donde planean ser utilizadas debido a la evidencia de diferencias en cuanto a los puntos de corte obtenidos respecto a los estudios originales.
